# (*E*)-*N*′-Benzyl­idene-5-methyl­isoxazole-4-carbohydrazide

**DOI:** 10.1107/S1600536809052969

**Published:** 2009-12-12

**Authors:** Yan-Xian Jin, Wen-Ping Jia, Jun-Yong Wu, Hua Yan

**Affiliations:** a School of Pharmaceutical and Chemical Engineering, Taizhou University, Linhai 317000, People’s Republic of China

## Abstract

The mol­ecule of the title compound, C_12_H_11_N_3_O_2_, is approximately planar with an r.m.s. deviation of 0.0814 Å from the plane through all the non-H atoms. The dihedral angle formed by the benzene and isoxazole rings is 6.88 (16)°. The mol­ecular conformation is stabilized by an intra­molecular C—H⋯N hydrogen bond, forming an *S*(6) ring, and the mol­ecule displays an *E* configuration with respect to the C=N double bond. In the crystal structure, inter­molecular N—H⋯O hydrogen bonds form centrosymmetric dimers which are further linked by weak C—H⋯N inter­actions augmented by very weak C—H⋯π contacts, forming layers parallel to (120).

## Related literature

For the biological activity and coordination ability of hydrazone compounds, see: Molina *et al.* (1994 ▶); Khattab (2005 ▶); Reiter *et al.* (1985 ▶). For the biological properties of isoxazole derivatives, see: Stevens & Albizati (1984 ▶). For related structures, see: Fun *et al.* (2008 ▶); Wei *et al.* (2009 ▶); Khaledi *et al.* (2008 ▶). For reference bond-length parameters, see: Allen *et al.* (1987 ▶). For hydrogen-bond motifs, see: Bernstein *et al.* (1995 ▶).
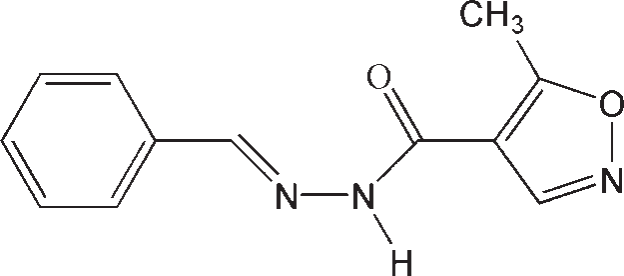


## Experimental

### 

#### Crystal data

C_12_H_11_N_3_O_2_
*M* *_r_* = 229.24
Triclinic, 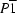

*a* = 6.6562 (6) Å
*b* = 7.4874 (9) Å
*c* = 11.3051 (11) Å
α = 87.319 (8)°
β = 84.640 (7)°
γ = 87.878 (8)°
*V* = 560.04 (10) Å^3^
*Z* = 2
Mo *K*α radiation
μ = 0.10 mm^−1^
*T* = 293 K
0.22 × 0.19 × 0.08 mm

#### Data collection

Bruker APEXII area-detector diffractometer
Absorption correction: multi-scan (*SADABS*; Bruker, 2004 ▶) *T*_min_ = 0.979, *T*_max_ = 0.992
6763 measured reflections
1936 independent reflections
1219 reflections with *I* > 2σ(*I*)
*R*_int_ = 0.040

#### Refinement

*R*[*F* ^2^ > 2σ(*F* ^2^)] = 0.095
*wR*(*F* ^2^) = 0.302
*S* = 1.10
1936 reflections
159 parameters
1 restraint
H atoms treated by a mixture of independent and constrained refinement
Δρ_max_ = 0.53 e Å^−3^
Δρ_min_ = −0.34 e Å^−3^

### 

Data collection: *APEX2* (Bruker, 2004 ▶); cell refinement: *SAINT* (Bruker, 2004 ▶); data reduction: *SAINT*; program(s) used to solve structure: *SHELXS97* (Sheldrick, 2008 ▶); program(s) used to refine structure: *SHELXL97* (Sheldrick, 2008 ▶); molecular graphics: *SHELXTL* (Sheldrick, 2008 ▶); software used to prepare material for publication: *SHELXL97*.

## Supplementary Material

Crystal structure: contains datablocks global, I. DOI: 10.1107/S1600536809052969/sj2706sup1.cif

Structure factors: contains datablocks I. DOI: 10.1107/S1600536809052969/sj2706Isup2.hkl

Additional supplementary materials:  crystallographic information; 3D view; checkCIF report

## Figures and Tables

**Table 1:** Hydrogen-bond geometry (Å, °)

*D*—H⋯*A*	*D*—H	H⋯*A*	*D*⋯*A*	*D*—H⋯*A*
C3—H3⋯N3	0.93	2.39	2.893 (5)	114
N2—H2⋯O2^i^	0.90 (1)	1.98 (1)	2.867 (4)	170 (4)
C1—H1*B*⋯N1^ii^	0.96	2.67	3.598 (6)	162
C1—H1*A*⋯*Cg*1^iii^	0.96	3.35	4.136 (7)	140

Symmetry codes: (i) 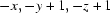; (ii) 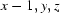; (iii) 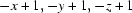. *Cg*1 is the centroid of the C7–C12 ring.

**Fig. 1.**
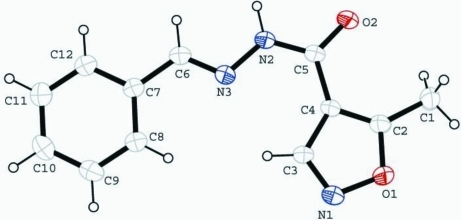
Molecular structure of of the title compound. Displacement ellipsoids are drawn at the 30% probability level.

**Fig. 2.**
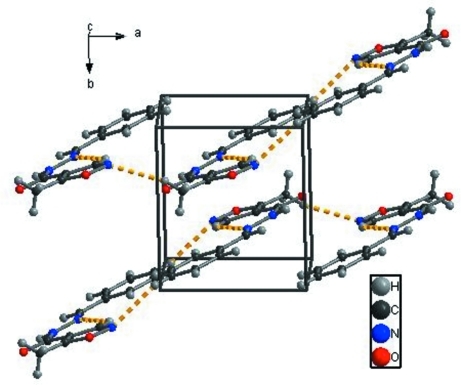
Packing diagram of the title compound. Intermolecular hydrogen bonds are shown as dashed lines.

## supplementary crystallographic information

### Comment

A large number of hydrazone derivatives have been reported recently (Fun *et al.* 2008; Wei *et al.* 2009; Khaledi *et al.* 2008) and their biological activity (Molina *et al.* 1994; Khattab 2005) and coordination ability (Reiter *et al.*1985) have also been noted. Isoxazole compounds have also attracted much interest as they exhibit some fungicidal, plant-growth regulating and antibacterial activity (Stevens *et al.*1984). In order to study the properties of a new compound containing both the hydrazine and isoxazole groups, we synthesized the title compound and report its crystal structure here, Fig 1.

The title compound C_12_H_11_N_3_O_2_, is approximately planar (rms deviation 0.0814 from the plane through all non-hydrogen atoms)with a dihedral angle of 6.88 (16)°. between the C7···C12 benzene and C2···C4,N1,O1 isoxazole rings. The molecule displays an E configuration with respect to the C6═N3 double bond, with a C7—C6—N3—N2 torsion angle of -179.3 (3)°. The dihedral angle formed by the benzene and isoxazole rings is 2.457 (114)°. An intramolecular C—H···N hydrogen bond generates an S6 ring motif (Bernstein *et al.* 1997) and locks the molecule into a planar configuration. Bond lengths (Allen *et al.*,1987) and angles are unexceptional and similar to those found in related stuctures (Fun *et al.*, 2008; Wei *et al.*, 2009; Khaledi *et al.*, 2008).

In the crystal structure (Fig. 2), intermolecular N2—H2···O2 hydrogen bonds form centrosymmetric dimers. These are further linked by weak C1—H1B···N1 interactions augmented by very weak, inversion related C1—H1A···Cg1 contacts to form layers parallel to [120] (Cg1 is the centroid of the C7···C12 phenyl ring).

### Experimental

Benzaldehyde (4.6 g,0.02 mol) and 5-methylisoxazole-4-carbonyl hydrazine (2.8 g, 0.02 mol) was mixed with glacial acetic acid (50 ml). The mixture was heated at 65° C for 3 h, the precipitate collected by filtration and washed with water, chloroform and ethanol. The product was recrystallized from ethanol, then dried under reduced pressure to give the title compound in 85% yield. Colourless, block-shaped crystals were obtained by slow evaporation of a dimethylformamide solution.

### Refinement

The H atom bound to N2 was located in a difference Fourier map and refined freely with the N–H distance restrained to 0.90 Å. All other H atoms were positioned geometrically and allowed to ride on their parent atoms, with C—H = 0.93–0.96 Å, and with *U*_iso_ = 1.2 *U*_iso_(C) or 1.5 *U*_iso_(C) for methyl groups.

### Crystal data

**Table:**

C_12_H_11_N_3_O_2_	*Z* = 2
*M**_r_* = 229.24	*F*(000) = 240
Triclinic, *P*1	*D*_x_ = 1.359 Mg m^−^^3^
Hall symbol: -P 1	Mo *K*α radiation, λ = 0.71073 Å
*a* = 6.6562 (6) Å	Cell parameters from 1711 reflections
*b* = 7.4874 (9) Å	θ = 2.7–23.4°
*c* = 11.3051 (11) Å	µ = 0.10 mm^−^^1^
α = 87.319 (8)°	*T* = 293 K
β = 84.640 (7)°	Block, colourless
γ = 87.878 (8)°	0.22 × 0.19 × 0.08 mm
*V* = 560.04 (10) Å^3^	

### Data collection

**Table:**

Bruker APEXII area-detector diffractometer	1936 independent reflections
Radiation source: fine-focus sealed tube	1219 reflections with *I* > 2σ(*I*)
graphite	*R*_int_ = 0.040
φ and ω scans	θ_max_ = 25.0°, θ_min_ = 1.8°
Absorption correction: multi-scan (*SADABS*; Bruker, 2004)	*h* = −7→7
*T*_min_ = 0.979, *T*_max_ = 0.992	*k* = −8→8
6763 measured reflections	*l* = −13→13

### Refinement

**Table:**

Refinement on *F*^2^	Primary atom site location: structure-invariant direct methods
Least-squares matrix: full	Secondary atom site location: difference Fourier map
*R*[*F*^2^ > 2σ(*F*^2^)] = 0.095	Hydrogen site location: inferred from neighbouring sites
*wR*(*F*^2^) = 0.302	H atoms treated by a mixture of independent and constrained refinement
*S* = 1.10	*w* = 1/[σ^2^(*F*_o_^2^) + (0.2*P*)^2^] where *P* = (*F*_o_^2^ + 2*F*_c_^2^)/3
1936 reflections	(Δ/σ)_max_ = 0.004
159 parameters	Δρ_max_ = 0.53 e Å^−^^3^
1 restraint	Δρ_min_ = −0.33 e Å^−^^3^

### Special details

**Table:**

Geometry. All e.s.d.'s (except the e.s.d. in the dihedral angle between two l.s. planes) are estimated using the full covariance matrix. The cell e.s.d.'s are taken into account individually in the estimation of e.s.d.'s in distances, angles and torsion angles; correlations between e.s.d.'s in cell parameters are only used when they are defined by crystal symmetry. An approximate (isotropic) treatment of cell e.s.d.'s is used for estimating e.s.d.'s involving l.s. planes.
Refinement. Refinement of *F*^2^ against ALL reflections. The weighted *R*-factor *wR* and goodness of fit *S* are based on *F*^2^, conventional *R*-factors *R* are based on *F*, with *F* set to zero for negative *F*^2^. The threshold expression of *F*^2^ > σ(*F*^2^) is used only for calculating *R*-factors(gt) *etc*. and is not relevant to the choice of reflections for refinement. *R*-factors based on *F*^2^ are statistically about twice as large as those based on *F*, and *R*- factors based on ALL data will be even larger.

### Fractional atomic coordinates and isotropic or equivalent isotropic displacement parameters (Å^2^)

**Table:**

	*x*	*y*	*z*	*U*_iso_*/*U*_eq_	
C1	0.1634 (6)	0.4088 (7)	0.0931 (4)	0.0719 (13)	
H1A	0.1434	0.5364	0.0932	0.108*	
H1B	0.0435	0.3528	0.1282	0.108*	
H1C	0.1922	0.3726	0.0128	0.108*	
C2	0.3348 (5)	0.3544 (5)	0.1625 (3)	0.0564 (11)	
C3	0.5697 (5)	0.2840 (6)	0.2794 (3)	0.0617 (11)	
H3	0.6344	0.2629	0.3484	0.074*	
C4	0.3642 (5)	0.3410 (5)	0.2793 (3)	0.0504 (10)	
C5	0.2072 (5)	0.3862 (5)	0.3748 (3)	0.0522 (10)	
C6	0.4198 (6)	0.2675 (5)	0.6388 (3)	0.0569 (10)	
H6	0.3092	0.3072	0.6882	0.068*	
C7	0.5963 (6)	0.1885 (5)	0.6923 (3)	0.0535 (10)	
C8	0.7688 (6)	0.1292 (6)	0.6238 (4)	0.0636 (11)	
H8	0.7727	0.1374	0.5413	0.076*	
C9	0.9325 (6)	0.0591 (6)	0.6767 (4)	0.0726 (13)	
H9	1.0466	0.0202	0.6299	0.087*	
C10	0.9299 (7)	0.0456 (6)	0.7985 (4)	0.0741 (13)	
H10	1.0417	−0.0017	0.8342	0.089*	
C11	0.7604 (7)	0.1027 (6)	0.8669 (4)	0.0785 (14)	
H11	0.7569	0.0934	0.9493	0.094*	
C12	0.5962 (7)	0.1736 (6)	0.8140 (4)	0.0684 (12)	
H12	0.4826	0.2122	0.8613	0.082*	
H2	0.150 (5)	0.408 (5)	0.545 (3)	0.075 (12)*	
N1	0.6576 (5)	0.2644 (5)	0.1742 (3)	0.0742 (12)	
N2	0.2397 (4)	0.3615 (4)	0.4894 (3)	0.0578 (9)	
N3	0.4137 (5)	0.2831 (4)	0.5270 (3)	0.0546 (9)	
O1	0.5060 (4)	0.3099 (4)	0.0977 (2)	0.0689 (10)	
O2	0.0417 (4)	0.4503 (4)	0.3494 (2)	0.0672 (9)	

### Atomic displacement parameters (Å^2^)

**Table:**

	*U*^11^	*U*^22^	*U*^33^	*U*^12^	*U*^13^	*U*^23^
C1	0.052 (2)	0.111 (3)	0.051 (2)	0.007 (2)	−0.0067 (19)	0.012 (2)
C2	0.039 (2)	0.072 (2)	0.054 (2)	0.0057 (17)	0.0052 (16)	0.0055 (18)
C3	0.041 (2)	0.095 (3)	0.046 (2)	0.0154 (19)	0.0029 (16)	−0.0016 (19)
C4	0.0349 (19)	0.065 (2)	0.049 (2)	0.0093 (15)	0.0018 (15)	0.0009 (16)
C5	0.037 (2)	0.068 (2)	0.051 (2)	0.0094 (16)	0.0003 (15)	−0.0050 (17)
C6	0.045 (2)	0.072 (2)	0.051 (2)	0.0075 (17)	0.0048 (17)	−0.0029 (17)
C7	0.050 (2)	0.062 (2)	0.047 (2)	0.0037 (17)	0.0017 (16)	−0.0022 (16)
C8	0.054 (2)	0.084 (3)	0.051 (2)	0.010 (2)	0.0017 (18)	−0.0024 (19)
C9	0.053 (2)	0.086 (3)	0.077 (3)	0.017 (2)	−0.002 (2)	−0.003 (2)
C10	0.066 (3)	0.080 (3)	0.078 (3)	0.015 (2)	−0.021 (2)	0.004 (2)
C11	0.078 (3)	0.099 (3)	0.057 (3)	0.020 (3)	−0.011 (2)	0.002 (2)
C12	0.065 (3)	0.087 (3)	0.052 (2)	0.016 (2)	0.0003 (19)	−0.004 (2)
N1	0.0411 (19)	0.121 (3)	0.057 (2)	0.0189 (18)	0.0007 (15)	0.0025 (19)
N2	0.0372 (17)	0.087 (2)	0.0468 (19)	0.0153 (15)	0.0002 (13)	−0.0059 (16)
N3	0.0408 (17)	0.073 (2)	0.0486 (19)	0.0090 (14)	−0.0024 (13)	−0.0020 (14)
O1	0.0457 (16)	0.113 (2)	0.0448 (16)	0.0136 (14)	0.0042 (12)	0.0022 (14)
O2	0.0419 (16)	0.104 (2)	0.0547 (17)	0.0250 (14)	−0.0063 (12)	−0.0128 (14)

### Geometric parameters (Å, °)

**Table:**

C1—C2	1.478 (5)	C7—C12	1.375 (5)
C1—H1A	0.9600	C7—C8	1.394 (5)
C1—H1B	0.9600	C8—C9	1.368 (5)
C1—H1C	0.9600	C8—H8	0.9300
C2—O1	1.337 (4)	C9—C10	1.374 (6)
C2—C4	1.352 (5)	C9—H9	0.9300
C3—N1	1.288 (5)	C10—C11	1.373 (6)
C3—C4	1.417 (5)	C10—H10	0.9300
C3—H3	0.9300	C11—C12	1.373 (6)
C4—C5	1.473 (5)	C11—H11	0.9300
C5—O2	1.239 (4)	C12—H12	0.9300
C5—N2	1.336 (5)	N1—O1	1.412 (4)
C6—N3	1.269 (5)	N2—N3	1.373 (4)
C6—C7	1.464 (5)	N2—H2	0.902 (10)
C6—H6	0.9300		
			
C2—C1—H1A	109.5	C8—C7—C6	122.2 (3)
C2—C1—H1B	109.5	C9—C8—C7	120.7 (4)
H1A—C1—H1B	109.5	C9—C8—H8	119.7
C2—C1—H1C	109.5	C7—C8—H8	119.7
H1A—C1—H1C	109.5	C8—C9—C10	120.6 (4)
H1B—C1—H1C	109.5	C8—C9—H9	119.7
O1—C2—C4	109.7 (3)	C10—C9—H9	119.7
O1—C2—C1	115.1 (3)	C11—C10—C9	119.2 (4)
C4—C2—C1	135.3 (4)	C11—C10—H10	120.4
N1—C3—C4	113.2 (3)	C9—C10—H10	120.4
N1—C3—H3	123.4	C12—C11—C10	120.3 (4)
C4—C3—H3	123.4	C12—C11—H11	119.9
C2—C4—C3	103.5 (3)	C10—C11—H11	119.9
C2—C4—C5	123.5 (3)	C11—C12—C7	121.3 (4)
C3—C4—C5	133.0 (3)	C11—C12—H12	119.4
O2—C5—N2	118.7 (3)	C7—C12—H12	119.4
O2—C5—C4	119.9 (3)	C3—N1—O1	104.4 (3)
N2—C5—C4	121.4 (3)	C5—N2—N3	123.3 (3)
N3—C6—C7	121.8 (3)	C5—N2—H2	119 (3)
N3—C6—H6	119.1	N3—N2—H2	118 (3)
C7—C6—H6	119.1	C6—N3—N2	115.5 (3)
C12—C7—C8	117.9 (4)	C2—O1—N1	109.3 (3)
C12—C7—C6	119.8 (4)		
			
O1—C2—C4—C3	0.0 (4)	C7—C8—C9—C10	0.0 (7)
C1—C2—C4—C3	−178.5 (5)	C8—C9—C10—C11	0.3 (7)
O1—C2—C4—C5	178.0 (3)	C9—C10—C11—C12	−0.4 (8)
C1—C2—C4—C5	−0.5 (8)	C10—C11—C12—C7	0.3 (8)
N1—C3—C4—C2	0.0 (5)	C8—C7—C12—C11	0.1 (7)
N1—C3—C4—C5	−177.7 (4)	C6—C7—C12—C11	−179.0 (4)
C2—C4—C5—O2	−4.5 (6)	C4—C3—N1—O1	0.0 (5)
C3—C4—C5—O2	172.8 (4)	O2—C5—N2—N3	176.6 (3)
C2—C4—C5—N2	176.3 (4)	C4—C5—N2—N3	−4.2 (6)
C3—C4—C5—N2	−6.4 (7)	C7—C6—N3—N2	−179.3 (3)
N3—C6—C7—C12	−179.8 (4)	C5—N2—N3—C6	−179.1 (4)
N3—C6—C7—C8	1.2 (6)	C4—C2—O1—N1	0.0 (5)
C12—C7—C8—C9	−0.2 (7)	C1—C2—O1—N1	178.8 (3)
C6—C7—C8—C9	178.8 (4)	C3—N1—O1—C2	0.0 (5)

### Hydrogen-bond geometry (Å, °)

**Table:**

Cg1 is the centroid of the C7–C12 ring.

**Table:**

*D*—H···*A*	*D*—H	H···*A*	*D*···*A*	*D*—H···*A*
C3—H3···N3	0.93	2.39	2.893 (5)	114
N2—H2···O2^i^	0.90 (1)	1.98 (1)	2.867 (4)	170 (4)
C1—H1B···N1^ii^	0.96	2.67	3.598 (6)	162
C1—H1A···Cg1^iii^	0.96	3.35	4.136 (7)	140

Symmetry codes: (i) −*x*, −*y*+1, −*z*+1; (ii) *x*−1, *y*, *z*; (iii) −*x*+1, −*y*+1, −*z*+1.

## References

[bb1] Allen, F. H., Kennard, O., Watson, D. G., Brammer, L., Orpen, A. G. & Taylor, R. (1987). *J. Chem. Soc. Perkin Trans. 2*, pp. S1–19.

[bb2] Bernstein, J., Davis, R. E., Shimoni, L. & Chang, N.-L. (1995). *Angew. Chem. Int. Ed. Engl.* **34**, 1555–1573.

[bb3] Bruker (2004). *APEX2*, *SAINT* and *SADABS* Bruker AXS Inc., Madison, Wisconsin, USA.

[bb4] Fun, H.-K., Patil, P. S., Rao, J. N., Kalluraya, B. & Chantrapromma, S. (2008). *Acta Cryst.* E**64**, o1707. 10.1107/S160053680802446X PMC2960631 21201695

[bb5] Khaledi, H., Mohd Ali, H. & Ng, S. W. (2008). *Acta Cryst.* E**64**, o2481. 10.1107/S1600536808039342 PMC2959793 21581446

[bb6] Khattab, S. N. (2005). *Molecules*, **10**, 1218–1228. 10.3390/10091218 PMC6147684 18007388

[bb7] Molina, P., Almendros, O. & Fresneda, P. M. (1994). *Tetrahedron*, **50**, 2241–2243.

[bb8] Reiter, J., Somoral, T. & Dvortsak, P. (1985). *Heterocycl. Chem.* **22**, 385–394.

[bb9] Sheldrick, G. M. (2008). *Acta Cryst.* A**64**, 112–122. 10.1107/S0108767307043930 18156677

[bb10] Stevens, R. V. & Albizati, K. F. (1984). *Tetrahedron Lett* **25**, 4587–4591.

[bb11] Wei, Y.-J., Wang, F.-W. & Zhu, Q.-Y. (2009). *Acta Cryst.* E**65**, o688. 10.1107/S1600536809007466 PMC2968809 21582430

